# Effectiveness of Negative Pressure Wound Therapy in Burns in Pediatric and Adolescent Patients: A Systematic Review and Meta-Analysis

**DOI:** 10.3390/healthcare14020242

**Published:** 2026-01-19

**Authors:** Celia Villalba-Aguilar, Juan Manuel Carmona-Torres, Lucía Villalba-Aguilar, Matilde Isabel Castillo-Hermoso, Rosa María Molina-Madueño, José Alberto Laredo-Aguilera

**Affiliations:** 1 Hospital Laboral Solimat, Calle San Pedro el Verde 35, 45004 Toledo, Spain; celia.villalba@alu.uclm.es; 2 Facultad de Fisioterapia y Enfermería de Toledo, Universidad de Castilla-La Mancha, Avda Carlos III s/n, 45071 Toledo, Spain; matildei.castillo@uclm.es (M.I.C.-H.); rosam.molina@uclm.es (R.M.M.-M.); josealberto.laredo@uclm.es (J.A.L.-A.); 3 Grupo de Investigación Multidisciplinar en Cuidados (IMCU), Universidad de Castilla-La Mancha, Avda. Carlos III s/n, 45071 Toledo, Spain; 4 Instituto de Investigación Sanitaria de Castilla-La Mancha (IDISCAM), Finca de la Peraleda s/n, 45004 Toledo, Spain; 5 Facultad de Ciencias de la Salud, Universidad Rey Juan Carlos, 28922 Alcorcón, Spain; luciavillalbaaguilar12@gmail.com; 6 Hospital Nacional de Parapléjicos, Finca de la Peraleda s/n, 45004 Toledo, Spain

**Keywords:** burn, child, negative pressure wound therapy, scar, graft, cost

## Abstract

**Background:** Burns represent a public health problem because they generate both physical and psychological damage, especially in the child and adolescent population, and high costs, especially due to the management of scars. Advances in burn care have improved survival and quality of life for this population. New clinical trials have been conducted on the benefits of negative pressure wound therapy (NPWT), showing that it improves the healing of burns and the appearance of scars. Therefore, this study aims to analyze the efficacy of NPWT both alone and as an adjunct to conventional dressings in pediatric and adolescent patients compared with conventional treatments. **Methodology**: A systematic search was carried out between December 2023 and the last quarter of 2025 in databases such as PubMed, Scopus, CINAHL, and the Cochrane Library. This meta-analysis was performed following the PRISMA statement (Preferred Reporting Items for Systematic Reviews and Meta-Analyses) and was registered in PROSPERO with registration number CRD42024597293. The risk of bias 2 (RoB2) tool was used to assess the risk of bias in the studies. Quantitative meta-analyses using random-model effects were performed only for variables with sufficient comparable data among studies. For other outcomes, where meta-analysis was not feasible due to lack of comparable data or control groups, results were synthesized qualitatively. **Results:** A total of seven articles (three clinical trials and four retrospective studies), in which a total of 323 subjects participated, were included. The main results demonstrate the efficacy of NPWT, as it decreases the re-epithelialization time, improves the appearance of scars (MD = −1.25 (95% CI between −1.80 and −0.70)), reduces the probability of skin grafts (OR = 0.17 (95% CI between 0.06 and 0.46)), and therefore, as there is less need for surgery and fewer dressing changes, reduces costs. **Conclusions**: NPWT offers significant clinical benefits in the treatment of burns in children and adolescents. Although a meta-analysis could not be performed due to the lack of a control group in some studies, studies with larger samples and multicenter designs will be necessary to better assess the relevant clinical outcomes. However, the results of this study show that NPWT is effective in treating burns in children and adolescents and that its use in clinical practice may represent a promising adjunctive therapy.

## 1. Introduction

Burns are tissue injuries caused by the transfer of energy from a body to the body [1] and are caused by causal agents such as heat, which is referred to as thermal burns, with 85% of cases normally occurring among children under 5 years of age; cold, although less frequent in children (2%) [2]; electricity, which causes high morbidity and mortality, although it is less frequent, with a 3% incidence in minors; heat, with 2% to 10%, which causes high functional and aesthetic morbidity; and solar or ionizing radiation [3,4], of which it is estimated that 50% of children experience at least one sunburn before the age of 11 [4,5].

Burn injuries are a major global public health problem [1] and generate, in addition to physical and psychological damage, large expenditures of resources to victims and their families [3]. A systematic review established a mean total cost of burn injuries in children of USD 3883 in high-income countries [6]. Nevertheless, the economic burden is likely to be considerably higher in low- and middle-income countries (LMICs). In these settings, limited access to specialized burn care, higher rates of infection and disability, and prolonged recovery periods significantly increase indirect costs and loss of productivity. Moreover, 90% of childhood burn deaths occur in LMICs, where prevention and rehabilitation programs are scarce [7]. In Spain, burns are an important cause of accidental morbidity and mortality in children and represent the fourth cause of accidental death in childhood, with a 33% incidence between 12 and 24 months of age [4].

Advances in burn care have improved the survival of children and adolescents. In addition, the main objective of caring for burns in this population is to prevent the formation of scars [8,9]. They can be functionally restrictive, aesthetically disfiguring, painful, and itchy, and have psychological consequences and even self-esteem problems [10]. Long re-epithelialization times, hospital admissions, and follow-up times can influence the appearance of hypertrophic scars [11]. These are raised, hyperpigmented, hard, thick scars with loss of elasticity above the level of the skin but that remain at the edges of the lesion [11,12]. Management costs range from USD 1000 to more than USD 10,000, depending on therapies used [13,14].

The most common pediatric treatment is topical antibiotics and antiseptics [15]. Other options include laser therapy and ultraviolet light to reduce scars; cultured skin cures and bioengineered therapies, which are very expensive [4]; stem cells and regenerative therapies, such as platelet-rich plasma (PRP) [16]; hydrogels [15,17]; and grafts or skin transplants, although the evidence is limited for this type of treatment [15,18].

Negative pressure wound therapy (NPWT) is worth highlighting because it seems to be a very effective technique in the care of burns in children, both physically and psychologically, as it is more profitable and even results in shorter hospital stays for patients with this type of wound [9,19]. This technique was approved by the Food and Drug Administration (FDA) in 1997 [20].

NPWT is a mechanical treatment that accelerates wound healing through the drainage of excess exudate and edema and the elimination of cell proliferation barriers [21,22]. Therefore, when NPWT is applied in pediatric patients, the negative pressure must be appropriate for their age, duration of treatment, frequency of dressing changes and location of the wound, and it is particularly indicated for small to moderate area burns (<10% total body surface area (TBSA)), burns in areas with difficult contour (hands, feet, perineum), or to secure grafts following surgical excision [9]. Even so, certain absolute contraindications for NPWT have been identified, including malignant wounds, necrotic tissue with eschar, or hypersensitivity to the therapy itself; and relative contraindications include exposed blood vessels or organs, exposed anastomotic sites, respiratory emergencies, or chest drains and full-thickness burns.

Despite the fact that there is a previous systematic review [9] on the efficacy of NPWT in treating burns in pediatric patients, it is necessary to update the knowledge and carry out new reviews because, in recent years, new clinical trials have been carried out on the effect of NPWT on the healing of burns in children and adolescents, which can offer greater certainty about its benefits. Similarly, to our knowledge, there are no previous reviews that have performed a quantitative analysis (meta-analysis) on the effectiveness of NPWT in treating burns in pediatric patients who objectively and quantitatively synthesize the available data. Therefore, the objective of this study was to analyze the effectiveness of NPWT versus standard treatment in pediatric and adolescent patients with burns.

## 2. Methods

### 2.1. Information Sources

A systematic search was carried out during December 2023 and the last quarter of 2025 in the following databases: PubMed, Scopus, CINAHL, and the Cochrane Library. In addition to electronic database searches, we also performed handsearching of the reference lists of included articles to identify additional relevant studies. Furthermore, gray literature sources, such as conference abstracts and institutional repositories, were screened to minimize the risk of publication bias and reduce the likelihood of missing eligible studies. The present systematic review with meta-analysis was carried out following the Preferred Reporting Items for Systematic Reviews and Meta-Analyses (PRISMA) statement [23] (File S1). In addition, this meta-analysis has been registered in Prospero with registration number CRD42024597293.

### 2.2. Search Strategy

For the search, the literature search strategy mentioned in Table 1 was used.

The formulation of the research question was developed based on the PICO question format according to Table 2.

### 2.3. Selection Criteria

The inclusion and exclusion criteria chosen for the preparation of this systematic review are described in Table 3.

### 2.4. Selection of Studies and Data Extraction

Two investigators jointly searched the aforementioned databases and compiled a list of articles. After the duplicate articles were filtered, a preliminary selection was made by reading the title and abstract of the articles. A new selection was made by reading the full texts of the articles, which were selected following the inclusion and exclusion criteria of the present systematic review.

If the opinions of these two investigators did not agree during the selection process, the third investigator was consulted independently. The data were collected during the reading of the full text, and the following information was extracted: study authors and publication date, country, type of study, number of participants, intervention, main results, conclusions, and quality.

### 2.5. Quality Assessment 

In accordance with the recommendations of the Cochrane Collaboration, the risk of bias 2 (RoB2) tool was used [24] to assess the risk of bias in the clinical trials [25,26] included in this meta-analysis. This tool is structured in a fixed set of bias domains, which focus on different aspects of the design, conduct and reporting of RCTs. The reliability of the results of an RCT depends on the degree to which potential sources of bias have been avoided; therefore, six aspects of bias are examined, including the randomization process, deviations from planned interventions, missing outcome data, outcome measurement, selection of reported outcomes, and overall bias. An algorithm proposes a judgment on the risk of bias that arises from each domain of the studies on the basis of the answers to the questions. This is evaluated through a Likert-type scale with three options (according to the criteria): “uncertain”, “low risk”, and “high risk” [27].

To assess the risk of bias of the retrospective studies, the Joanna Briggs Institute (JBI) scale was used. This consists of 10 or 11 items (depending on the type of scale) that evaluate different key methodological aspects that ensure the quality of the study. This is evaluated through a dichotomous scale “yes” and “no”, and to allow greater flexibility when it is not possible to judge the fulfillment of an item definitively, or if it is not applied in the context of the evaluated study, the following are used: “unclear” or “not applicable” [28].

### 2.6. Statistical Analysis

A qualitative synthesis was carried out for each of the studies included in this systematic review. The data analyzed included the re-epithelialization time, the characteristics of the scar, the need for grafts, the presence of adverse effects and the cost-effectiveness.

For the quantitative analysis, a meta-analysis was performed. For the quantitative variables, the weighted mean differences were calculated before and after the intervention, together with their respective 95% confidence intervals (CIs). For binary variables, the odds ratio (OR) with its 95% CI was used. The results are represented by forest diagrams, establishing a level of statistical significance of α = 0.05.

Statistical heterogeneity between the studies was evaluated via the I^2^ statistic, which was classified as low (I^2^ ≤ 25%), moderate (I^2^ between 26% and 50%), or high (I^2^ ≥ 51%). The results are expressed with their respective 95% CIs. We prespecified the use of a random-effects model because the included trials differed in populations and settings, and we therefore assumed that the true intervention effect could vary across studies. The choice between fixed-effect and random-effects models was based on these conceptual assumptions rather than on the magnitude or statistical significance of heterogeneity, in line with standard recommendations. Given that only three studies were available, random-effects estimates were interpreted with caution because the between-study variance is imprecisely estimated when few studies are included. Therefore, we repeated all analyses under a fixed-effect model as a sensitivity analysis, which yielded similar results [29]. Publication bias was examined by visual inspection of the funnel plots generated for each meta-analysis. All the statistical estimates and analyses were performed via the Cochrane Collaboration’s RevMan software (version 5.4).

## 3. Results

### 3.1. Characteristics of the Included Results

**Figure 1:**
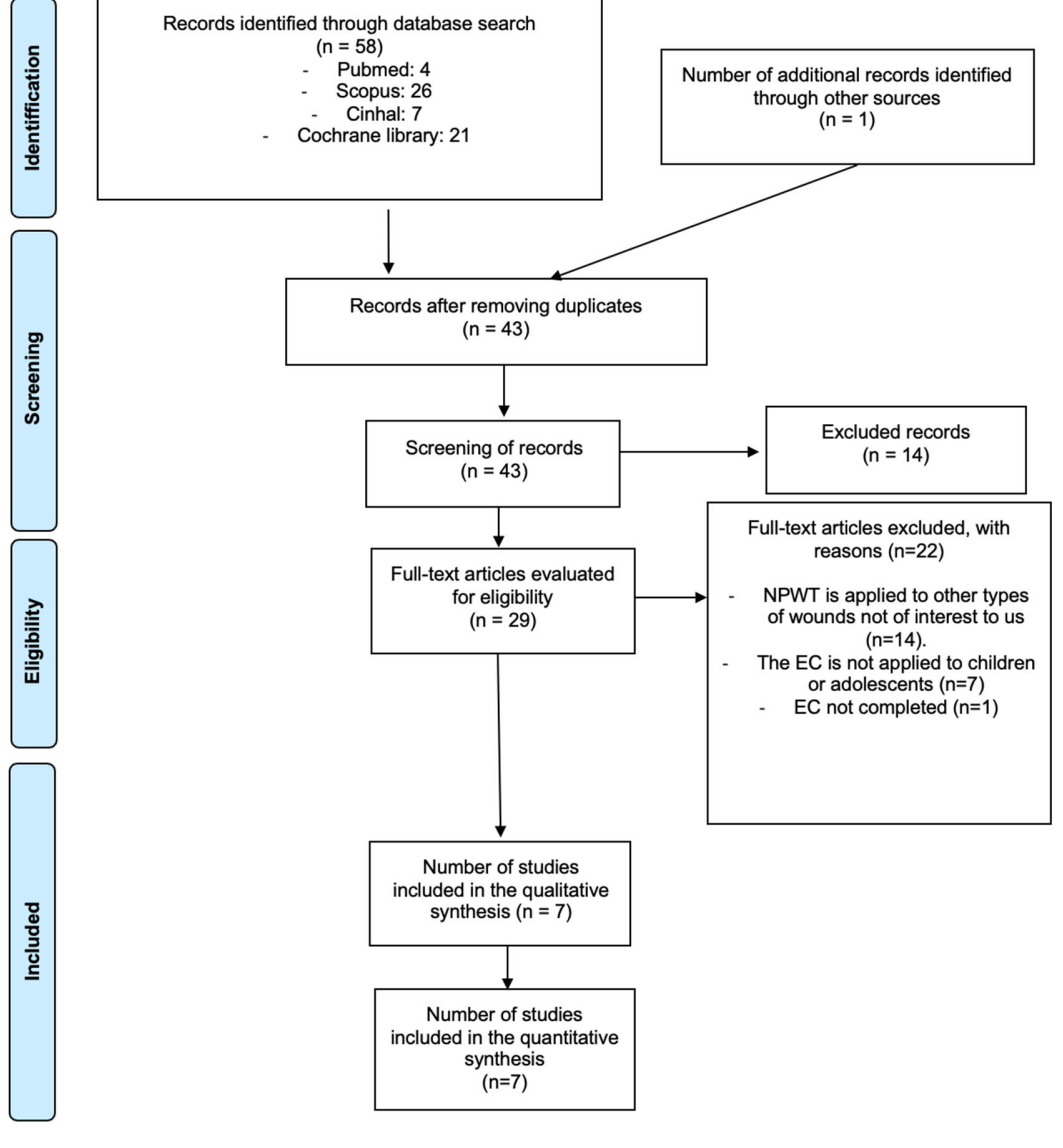
PRISMA flow diagram.

As shown in Figure 1, 58 articles extracted from the aforementioned databases were initially analyzed. A total of 15 duplicate articles were subsequently eliminated through the Mendeley bibliographic manager, and a screening was carried out, first by title and abstract, where 14 articles were excluded; later, the 22 full-text articles were excluded for the reasons explained in Figure 1, leaving a total of 7 articles.

Therefore, a total of seven articles (Table 4) met the inclusion and exclusion criteria for inclusion in the research, with a sample size of 323 participants. All participants were under 18 years of age, except for one 24-year-old patient [29] who presented with some burns; 217 patients were treated with NPWT and 106 were treated with standard dressings.

Table 4 describes the main characteristics of the seven studies included in the review, observing that they have been carried out in different countries (two in Australia, both in Queensland [25,26], two in the USA [31,32], two in China [33,34], and one in Graz, Austria [30]), these studies demonstrate the different durations of the intervention and the follow-up periods, which is why variability in different populations and settings will occur. The number of participants in each study ranged between 3 and 101. The duration of the intervention ranged between 8 and 90 days.

Table 5 shows the demographic characteristics of the participants, as well as the type of wound and the characteristics of the burn. The ages of the participants ranged from 2 months to 18 years, except for one subject who was 24 years old [30]. There was a higher percentage of boys than girls in all the articles included. All participants presented burns from different causative agents: scalds [25,26,30,31,33], contact [25,26,30,31], flame [25,26,30,31], electrical [31], burning clothes [31], freezing [32], and chemical and air stream [29]. Others also experience abrasions from traffic accidents [33] and Stevens–Johnson syndrome [31]. Three articles included <6% of TBSA [25,26,30,34], and one article included between 27.62% and 9.83% of the TBSA [31].

Table 6 summarizes the use of the NPWT in the different studies. Two types of NPWTs are used: the Renasys Touch^™^ NPWT (Smith and Nephew) and the KCI VAC^®^. Negative pressures between −40 mmHg (when patients are younger than 12 months to reduce ischemic damage) and −150 mmHg were used. Additional materials used alone or in combination with NPWT include silver impregnated mesh (Acticoat^TM^) and Mepitel^TM^ silicone or silver sulfadiazine cream dressing.

### 3.2. Risk of Bias Assessment

**Figure 2:**
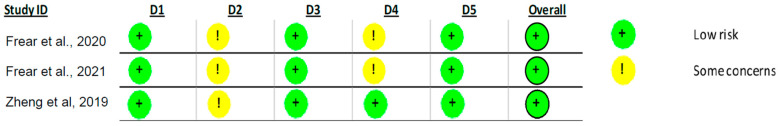
Assessment table of biases in literature of included clinical trials [25,26,34].

**Figure 3:**
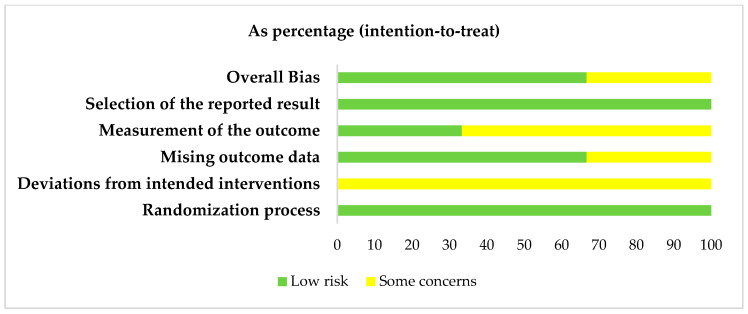
Overall risk of bias in clinical trials.

According to the Rob2 tool for systematic reviews of interventions, the bias of the two included clinical trials was assessed [25,26,34]. These are shown in Figure 2 and Figure 3. The three clinical trials had overall low bias scores, and key evaluations, such as re-epithelialization, were performed in a blinded manner, which strengthens the validity of the results, although there are certain doubts or possible identified risks in terms of masking in participants and staff.

In the case of the studies by Hoeller et al. [30], Ren et al. [31], and Poulakidas et al. [32], the JBI scale was used for the case series [35], and for the article by Yuan et al. [33], the JBI scale was used for cases and controls [36]. Articles with fewer than six positive items (“yes”) were excluded from our review. None of the studies were excluded because of their quality.

**Figure 4:**
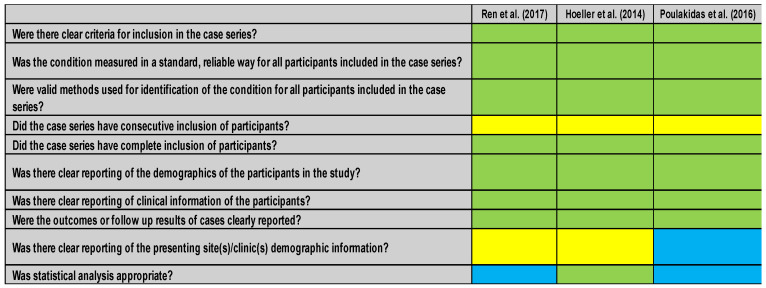
JBI for case series studies. Green is [Yes], yellow is [Unclear], and blue is [Not applicable] [30,31,32].

The results of the case series studies [30,31,32] are shown in Figure 4. These studies show variations in terms of inclusion criteria, demographic data, and statistical analysis. All of them describe the inclusion criteria and clinical data of the patients but do not clarify whether the inclusion criteria were consecutive or provide demographic information on the place of treatment. Others include adequate statistical analysis [30], but neither does the consecutive character of the inclusion. Likewise, the demographic data concerning the sites of care were incomplete.

**Figure 5:**
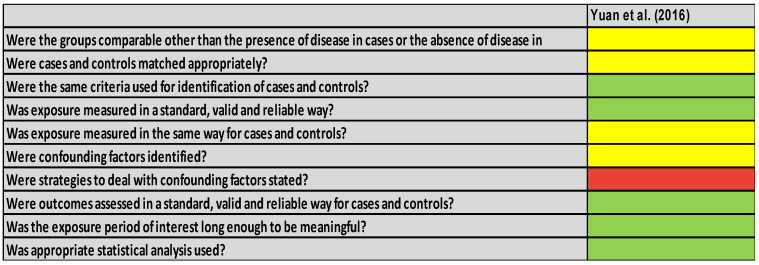
JBI for case-control study of Yuan et al. [33]. Green is [Yes], red is [No], yellow is [Unclear].

For the case-control study [33], the results are shown in Figure 5. The study does not specifically mention the comparability of the groups apart from the presence of burns, nor does it mention a specific pairing of cases and controls in the study. However, exposure and outcomes were measured in a standard way, suggesting a valid and reliable measurement, although it does not explicitly mention confounding factors or strategies to manage them. Thus, an appropriate statistical analysis was used.

Overall, the RoB2 assessments (Figure 2 and Figure 3) indicated that the two randomized controlled trials were at low overall risk of bias [25,26], particularly in outcome measurement and reporting, which increases confidence in their findings. In contrast, the JBI assessments (Figure 4 and Figure 5) showed that the retrospective studies [30,31,32] had methodological limitations, such as incomplete demographic reporting and unclear recruitment procedures, which may reduce the certainty of their results. These differences in study quality should be considered when interpreting the pooled evidence.

### 3.3. Re-Epithelialization Time

Of the seven articles included in the review, three addressed the re-epithelialization time [25,31,33]. In accordance with Frear CC, et al. [25] 95% of the participants achieved spontaneous re-epithelialization, and furthermore, the mean time to total re-epithelialization was 10 days in the CG compared with 8 days in the IG. In two articles, it was observed that with NPWT, there was faster granulation tissue formation [31,33]. In the study of Zheng et al., the complete wound healing time of patients in IG was significantly shorter than that in the CG (*p* < 0.01) [34]. Therefore, it seems that, in the included articles, the time of granulation tissue formation was shorter in the patients treated with NPWT.

### 3.4. Scar

Of the seven articles included in the review, three address the aspect of scars [25,33,34]. In accordance with Frear et al. [25], the risk of referral to scar treatment was 60% lower in the IG than in the CG (*p* = 0.013). In this study, validated instruments such as the Patient and Observer Scar Assessment Scale (POSAS) and the Brisbane Burn Scar Impact Profile (BBSIP) were used, in addition to ultrasound and colorimetry, and the questionnaires demonstrated a better perception of scar severity by caregiver in the IG. The scar outcomes of Yuan et al. [32] and Zheng et al. [34] were assessed using the validated Vancouver Scar Assessment Scale (VSS), which demonstrated significantly lower scores in the intervention group compared with controls, indicating lighter and more elastic scars. Nevertheless, different validated tools were applied across studies (POSAS/BBSIP vs. VSS), direct comparability of scar outcomes is limited.

**Figure 6:**
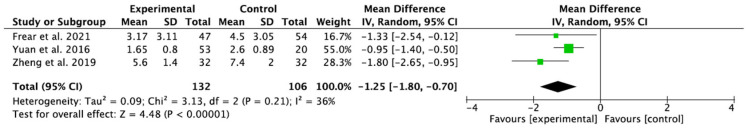
Forest plot of scar [25,33,34].

**Figure 7:**
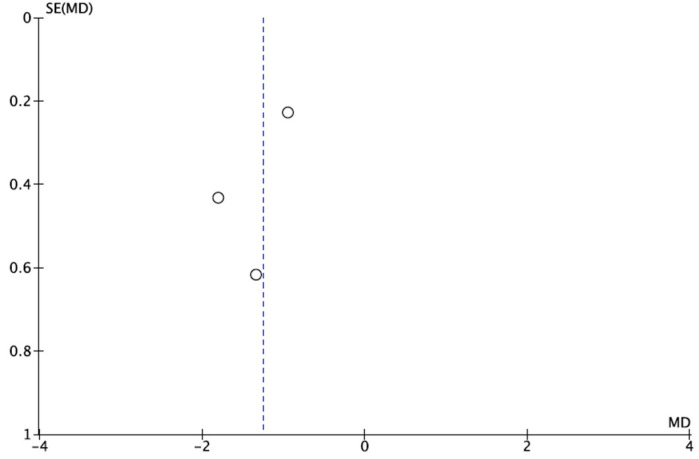
Funnel plot of scar [25,33,34].

The results of the meta-analysis in terms of scars are shown in Figure 6. Compared with conventional dressings, treatment with NPWT significantly reduced the scar appearance, with a MD = −1.25 (95% CI between −1.80 and −0.70). The heterogeneity (I^2^ = 36%) strengthens the confidence in the results (*p* < 0.00001). The risk of publication bias was defined by visual inspection of the funnel plot (Figure 7), which revealed that there appeared to be reasonable symmetry around the centerline, suggesting a low probability of publication bias.

### 3.5. Grafts

**Figure 8:**
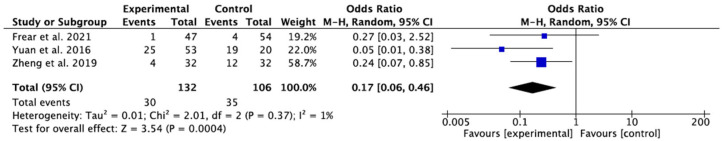
Forest plot of need for split-thickness skin grafts [25,33,34].

**Figure 9:**
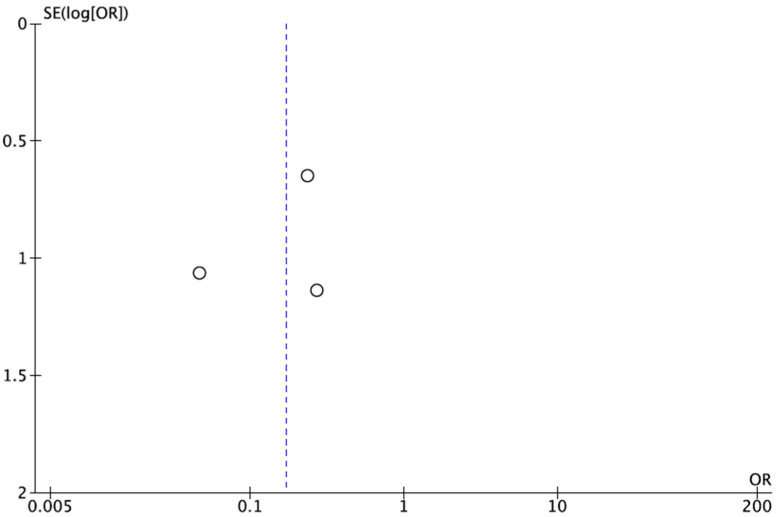
Funnel plot of split-thickness skin grafts [25,33,34].

Of the seven articles included in the review, three described a lower need for partial-thickness skin grafts [25,33,34]. The results of the meta-analysis regarding the need for skin grafts are shown in Figure 8. Compared with conventional dressings, treatment with NPWT significantly reduced the probability of partial-thickness skin grafts, with an OR = 0.17 (95% CI between 0.06 and 0.46). The lower heterogeneity (I^2^ = 1%) strengthens the robustness of the results (*p* = 0004). The risk of publication bias was defined by visual inspection of the funnel plot (Figure 9), which revealed that there appeared to be reasonable symmetry around the centerline, suggesting a low probability of publication bias.

### 3.6. Adverse Effects

All the articles [25,26,30,31,32,33,34] described and compared the possible adverse effects of both NPWT and conventional dressings. In accordance with Frear et al. [25], there were no statistically significant differences in terms of pain or itching between groups, while Yuan et al. [33] reported a decreased in these symptoms decreased with increasing NPWT. Both Ren et al. [31] and Yuan et al. [33] observed no bleeding events in patients treated with NPWT. With respect to infections, Yuan et al. [32] reported bacterial detection (without distinction between colonization and clinical infection) in 51 of 53 patients in the IG, most commonly *Enterobacter cloacae*, *E. coli*, *Staphylococcus epidermidis*, *P. aeruginosa*, and *A. baumannii*. Despite the high rate of bacterial growth, sequential therapy effectively controlled these cases, achieving graft success in 50 of 53 patients. These graft losses were associated with infection, two by *P. aeruginosa* and one by *A. baumannii*. In the CG, bacteria were detected in 17 cases, including *E. coli*, *Enterococcus faecium*, *S. epidermis*, *P. aeruginosa*, and *A. baumannii*. Supporting these findings, Zheng et al. reported significantly lower bacterial positivity rates in IG compared to conventional treatment: 31.25% and 18.75% at days 14 and 21 post-injury, respectively, versus 62.50% and 40.62% in the CG (*p* < 0.01) [34]. In contrast, Frear et al. [25] reported no infections in either group, whereas Hoeller et al. [30] observed a mild local infection and a NPWT system was contaminated with stool due to installation in the anogenital region. It is important to note that the definition of “infection” was not standardized across studies. Yuan et al. [33] reported bacterial culture positivity, which may reflect colonization rather than clinical infection, whereas Frear et al. [25] reported no infections based on clinical diagnostic criteria. This inconsistency prevents direct cross-study comparison of infection-related outcomes.

**Figure 10:**
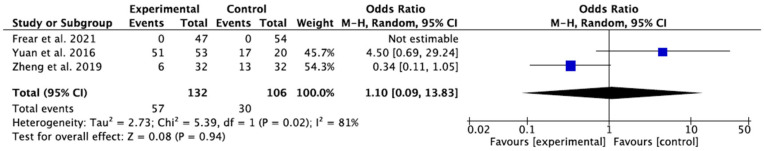
Forest plot of infections [25,33,34].

The results of the meta-analysis regarding infections are shown in Figure 10. Three studies were included [25,33,34]. The pooled analysis yielded an OR = 1.00 with a 95% confidence interval ranging from 0.13 to 8.03, indicating no statistically significant difference between the experimental and control groups (*p* = 0.94). The study by Frear et al., was not estimable due to zero events in the experimental group. Importantly, the analysis revealed substantial heterogeneity (I^2^ = 81%, *p* = 0.02), suggesting considerable variation across studies [25]. Despite the lack of statistical significance, the presence of heterogeneity highlights the need for cautious interpretation and may reflect differences in study design, populations, or interventions.

**Figure 11:**
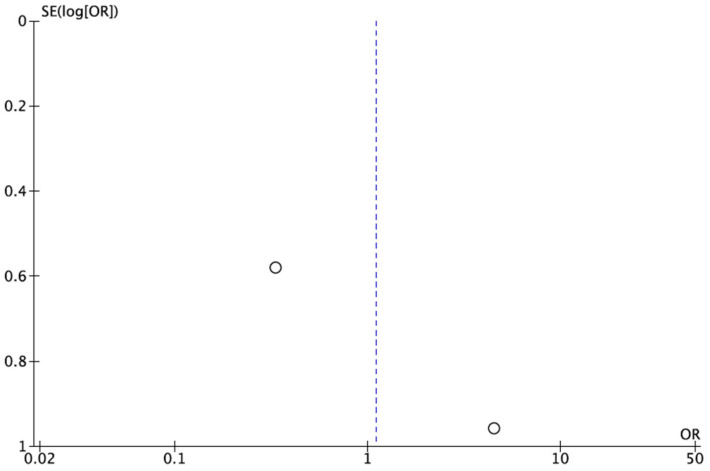
Funnel plot of infections [25,33,34].

The risk of publication bias was defined by visual inspection of the funnel plot (Figure 11); although the distribution of points appears slightly asymmetric, the small number of studies and the high heterogeneity (I^2^ = 81%) prevent any firm conclusions regarding publication bias.

Frear et al. [26] noted that two participants from the IG required additional surgery compared to seven in the CG, similar to Ren et al., where eight patients required reoperation [31]. Regarding treatment management, caregivers initially rated NPWT dressing changes as more difficult; however, the self-reports of the children did not reveal statistically significant differences in their management or movement. Hoeller et al. also observed NPWT patients demonstrated greater mobility compared to those receiving conventional treatments [30]. Notably, the patients of Poulakidas et al. with frostbite treated with NPWT did not require amputations [32].

### 3.7. Cost-Effectiveness

The cost-effectiveness analysis was performed in a single article [26] of the seven, indicating that the average total cost for the CG was greater than that for the IG. Likewise, Ren et al. [31] observed a lower need for dressing changes with NPWT, demonstrating a lower cost in terms of dressings. In terms of adverse effects, there was less need for surgery in the IG than in the CG [25], and the costs in this group were reduced.

## 4. Discussion

The objective of this review was to determine the efficacy of NPWT in treating burns in pediatric patients, both alone and as an adjunct to conventional dressings. The results of this study suggest that NPWT facilitates the formation of granulation tissue, reducing the time needed for the formation of epithelialized tissue, resulting in a better appearance of scars and reducing costs. In the articles used in this review, NPWT was used continuously from −40 mmHg (when patients were younger than 12 months) to −150 mmHg. The lack of uniformity in NPWT application across studies. Pressure settings varied widely (from −40 to −150 mmHg), and different commercial devices were used (e.g., Renasys Touch™ [25] vs. KCI VAC^®^ [30,31]). Such protocol variability may have influenced treatment outcomes, yet these factors were not examined as potential effect modifiers in the present review. Likewise, this therapy was used both on grafted skin [29] and in patients who did not require skin grafts, with the intention of promoting primary healing [31].

The results revealed a shorter re-epithelialization time in patients treated with NPWT than in those treated with conventional dressings [25,31,33,34]. Several studies have shown that this therapy results in 100% granulation tissue in a high percentage of adult patients and that the wounds close in less time than conventional dressings [37,38,39,40,41]. Consistently, Zheng et al. reported a shorter mean of complete wound healing time in the NPWT group compared to the CG [34].

There are several methods for strengthening skin grafts to improve their ability to adhere correctly [42]. However, compared with conventional dressings, NPWT has been shown to decrease the likelihood of partial-thickness skin grafts [25,33,34]. Hoeller et al. reported a pediatric patient in which, despite the use of NPWT on the graft tissue, most of the graft was lost; seven patients had peripheral necrosis, one patient had slight wound dehiscence, and another patient had graft displacement. However, the overall skin graft rate in their study was 96% [30]. Therefore, it is evident that NPWT facilitates the formation of granulation tissue and reduces the need for grafts by promoting local blood flow, eliminating excessive fluids and inflammatory mediators, and reducing the bacterial load [42,43]. Similarly, several authors consider NPWT to be a relevant option for treating burns in complicated anatomical areas with uneven contours, such as the hands, feet, genitals, and perianal regions [9,32].

The risk of referral to scar treatment was lower in patients treated with NPWT; likewise, NPWT favored the appearance of scars in terms of pigmentation and erythema [25,33,34], although there were no statistically significant differences in terms of thickness after 6 months [25]. However, laser therapies can be safe, effective, and tolerable options for the treatment of scars in this age group, eliminating the need for general anesthesia, as demonstrated by Roohaninasab et al. in their systematic review [44]. In our study, meta-analytic results showed moderate heterogeneity (I^2^ = 36%), indicating that while some variability exists between studies, the overall effect of NPWT on scar improvement is consistent and reliable. Validated assessment tools, including POSAS (V2.0, Beverwijk, The Netherlands), BBSIP (Child 8–18 years version; Brisbane, Queensland, Australia), and VSS, consistently showed that scars in intervention groups were perceived as less severe, lighter, and more elastic. Nevertheless, differences in assessment instruments across studies limit direct comparability of outcomes.

Adverse effects of NPWT are generally rare. However, interpretation of infection-related outcomes among the included studies should be made with caution, as not all authors distinguished between bacterial colonization and clinical infection. Yuan et al. [33] reported bacterial detection in most patients of the IG, but this was based on culture positivity rather than clinical diagnostic criteria. In contrast, Frear et al. observed no infections using clinical definitions [25], while Zheng et al. reported lower bacterial positivity rates with NPWT compared to conventional dressings [34]. The meta-analysis revealed no statistically significant difference in infection rates, but substantial heterogeneity (I^2^ = 81%) indicates considerable variability across studies, likely due to differences in study design, patient populations, infection definitions, and NPWT protocols. This highlights the need for standardized criteria for infection and colonization in future research. Pain, itching, bleeding, local infections, and problems with the NPWT device were the most common [45]. Pain assessment during changes was reported using validated pediatric pain scales such as the Wong–Baker faces pain rating scale and visual analog scales [25]. There are reports of high levels of pain associated with such therapy, especially when the negative pressure is greater than 125 mmHg [45]. Therefore, the pressure management of pediatric patients is more careful, and because dressing changes are painful, NPWT is more beneficial for the pediatric population, as demonstrated in the study by Kasukurthi et al. [42]. Younger children often have more difficulty with NPWT; therefore, according to Sahin et al., portable NPWT allows these children to move more easily [46], which makes them have better mobility than conventional dressings [30]. The price of negative pressure devices differs internationally. According to two retrospective studies, VAC is considered one of the more cost-effective therapies for treating burns or chronic wounds in the United States, although it appears to be more affordable in China [47,48]. Because this therapy can be used on an outpatient basis, it decreases the hospital stay; thus, it requires less need for dressing changes, and, therefore, the costs are lower [26,31]. Rai et al. observed that the mean length of hospital stay for patients with chronic wounds was 28.25 days with NPWT versus 39.17 days with saline dressings [49], as did Johari et al. in their trial; they also reported that NPWT decreased the need for hospitalization and the length of hospital stay due to the possibility of following outpatient treatment [50]. Apelqvist et al. found that the direct economic cost of the use of VAC dressings was greater than that of conventional dressings [51], which is consistent with the results of our study. Only one study included in this review provides evidence for cost-effectiveness and may vary across healthcare settings; nonetheless, the available data consistently suggest that NPWT may reduce hospital stay and the need for dressing changes, highlighting its potential economic benefit and the need for further research. Although NPWT may help lower medical costs, the most effective way to reduce hospitalization expenses for pediatric burns is still to prevent such injuries from occurring. Ensuring that children receive prompt and appropriate care after a burn, minimizing the length of hospital stays, and ultimately avoiding burn accidents altogether are key to reducing the overall medical burden [52].

### Limitations

It was not possible to perform a quantitative meta-analysis of all the observed outcomes because most of the included publications lacked a control group, as they were retrospective observational studies. Consequently, the quantitative synthesis could only be performed for three outcomes (scar pigmentation, scar erythema, and the need for skin grafts) and included only two studies per analysis, limiting the ability to generalize the results, and increasing the potential impact of publication bias.

Although the moderate or low levels of statistical heterogeneity appears favorable for scars and split-thickness skin grafts, the small number of included studies limits the sensitivity of this analysis, and true differences may remain undetected. Similarly, formal assessments of publication bias (e.g., Egger’s test) were not feasible due to the limited number of studies per outcome, so the possibility of unpublished negative results or selective reporting cannot be excluded.

Another relevant limitation is the overreliance on retrospective data. Four of the seven studies included in this review were retrospective (case series or case–control studies). Such designs are inherently more vulnerable to selection bias, unmeasured confounding variables, and inconsistent data collection methods, which may compromise internal validity. As a result, causal inferences drawn from these findings should be interpreted with caution, as retrospective evidence provides a lower level of certainty compared to prospective randomized controlled trials.

Additional methodological limitations were: first, definitions of “infection” were not standardized across studies: some authors reported bacterial colonization based on culture results, while others referred only to clinically diagnosed infections, limiting the validity of cross-study comparisons; second, scar outcomes were assessed with different tools: Frear et al. [25] used validated instruments such as POSAS and BBSIP, whereas Yuan et al. [33] and Zheng et al. [34] applied the Vancouver Scar Scale (VSS). This heterogeneity in outcome measurement reduces comparability and weakens the strength of pooled conclusions. Third, long-term scar outcomes could not be evaluated, as follow-up in the included studies was limited to 3–6 months, preventing conclusions about the durability of observed benefits.

Another limitation concerns the geographic and clinical homogeneity of the included studies. Most were conducted in high-income countries (USA [31,32], Australia [25,26], Austria [30]), with two from a middle-income country (China [33,34]). This limited geographical distribution restricts the global applicability of the results, as it may not reflect the challenges, healthcare resources, and outcomes in low- and middle-income countries (LMICS), where approximately 90% of childhood burn deaths occur. Future studies should therefore aim to include diverse settings, particularly LMICs, to improve the external validity and global relevance of findings regarding NPWT in pediatrics burn care.

In addition, most included studies are retrospective series or case reports, with very few randomized or prospective controlled trials. This inherently limits the strength of any pooled estimates and the confidence with which conclusions can be drawn. Moreover, the small number of studies (n = 7) and the marked variability in their sample sizes from as few as 3 participants [32] to 101 [25,26], represent an additional limitation. This heterogeneity may reduce the overall statistical power of the meta-analysis and limit the generalizability of the findings. Therefore, the results of this review should be interpreted with caution. There is a clear need for future large, multicenter, randomized controlled trials with standardized outcome measures to provide stronger evidence and enhance the reliability of conclusions regarding the effectiveness of NPWT in pediatric burn care.

## 5. Conclusions

The available evidence from the six studies included in this review suggests that negative pressure wound therapy (NPWT) shows potential clinical benefits for the management of burns in children and adolescents, either as an adjunct to conventional dressing or as a stand-alone treatment. Reported outcomes include accelerated granulation tissue formation and favorable scar appearance, with some reports of fewer dressing changes and possible cost savings.

However, these findings are derived primarily from small, retrospective studies and a limited number of comparative trials, which substantially limits the strength and generalizability of the conclusions. Larger, well-designed, multicenter randomized studies are required to confirm these preliminary observations and to more clearly define the role of NPWT in pediatric burn care.

## Figures and Tables

**Table 1:** Search strategy used for each database.

Database	Search Strategy
PubMed	(((“Child” [Mesh]) OR “Adolescent” [Mesh]) AND “Burns” [Mesh]) AND “Negative Pressure Wound Therapy” [Mesh] Filters: Clinical Trial, 2014–2025
Scopus	(TITLE (vacuum assisted closure) OR TITLE (negative pressure wound therapy) AND TITLE-ABS-KEY (child) OR TITLE-ABS-KEY (adolescent) AND TITLE-ABS-KEY (burn)) AND PUBYEAR > 2013 AND PUBYEAR < 2026 AND (LIMIT-TO (DOCTYPE, “ar”))
CINAHL	XB (children or adolescents or youth or child or teenager) AND TI (negative pressure wound therapy or npwt or vacuum assisted closure or wound vac) AND XB (burns or burn injury or burns trauma or major burns) Limiters: 2014–2025
Cochrane Library	(“negative pressure wound therapy” OR “npwt” OR “vacuum assisted closure” OR “wound vac” OR “vac therapy”) AND (“children” OR “adolescents” OR “youth” OR “child” OR “teenager” OR “pediatric” OR “paediatric” OR “kids” OR “infants”) AND (“burns” OR “burn injury” OR “burns trauma” OR “major burns” OR “burned limb”) Limits: 2014–2025

**Table 2:** Research questions in PICO format.

How Effective Is NPWT Treatment for Pediatric Burns?			
Population (P)	Intervention (I)	Comparison (C)	Outcomes (O)
Pediatric and adolescent patients under 24 years of age.	Use of NPWT in isolation or as an adjunct to Acticoat™, Mepitel™, or silver dressings in burns.	Conventional treatment based on conventional dressings (saline, wet, alginates, and silver sulfadiazine ointments).	Efficacy of negative pressure therapy measured by: objective criteria included time to re-epithelialization, presence of granulation tissue, need for grafts, and occurrence of adverse effects; and subjective criteria included pain, itching, and caregiver perception of scar appearance, as reported in the included studies.

**Table 3:** Inclusion and exclusion criteria.

Inclusion Criteria	Criteria Exclusion
Clinical trials and retrospective studies. Patients with burns. Population under 24 years of age.	Use of the NPWT in other types of interventions beyond our interest. Studies conducted on animals. Studies published over 11 years old.

**Table 4:** Main characteristics of studies included in the review.

Authors and Country	Participants	Type of Study	Intervention	Results	Conclusions	Risk of Bias
Hoeller et al., 2014 [30], Austria	N: 53	Retrospective single-center case series study	All wounds were initially cleaned, debrided and covered with silver dressings. NPWT with polyurethane foam was used, with continuous negative pressure from 70 to 125 mmHg depending on the age of the patients.	Average NPWT duration: 5 days (IQR 5–6).	NPWT is a constant, well implemented and useful tool to ensure the fixation of grafts to the skin. The main advantage is that mobility is much better compared to traditional methods.	Low
				Spearman correlation coefficient: higher rate of insertion tissue with NPWT, 96% skin graft acceptance rate. One case lost most of the graft.		
Ren et al., 2017 [31], USA	N: 29	Retrospective case series study in a single center	NPWT was not started until all necrotic tissue was removed from the wounds by surgical excision.	No bleeding with NPWT.	NPWT is safe and effective for children with localized complex burns. The benefits included fewer dressing changes, faster granulation, bleeding, and fewer days of hospitalization.	Low
			Negative pressure continued between 50 and 125 mmHg, and younger children received less negative pressure.	Eight patients had to go to the operating room.		
			The therapy dressings were changed every 5–7 days in the operating room.	Less need for dressing changes with NPWT.		
				Average length of hospital stay: 44 days.		
Frear et al., 2020 [25], Australia	N:101	ECA	The IG was treated with standard dressings consisting of Acticoat™ and Mepitel™ in combination with NPWT.	*Average re-epithelialization time:*	NPWT accelerated re-epithelialization in children with partial-thickness thermal burns of less than 5% of TBSA. In addition, it caused a decrease in the time expected for wound closure. NPWT did not cause pain and had positive effects on blood flow and histological neovascularization, as well as a decrease in the need for graft compared to SLN. Although six children experienced small blisters or macerations around the wound, a decrease in the thickness of the scar was also observed with this therapy.	Low
				IG: 8 days (IQR 7–11)		
	IG: 47		NPWT → −80 mmHg.	CG: 10 days (IQR 8–14)		
				*95% complete re-epithelialization.*		
			NPWT → −40 mmHg (extremities children under 12 months).	*Grafts:*		
				IG: 1		
	CG: 54		The SLN was treated with standard dressings only.	CG: 4		
				*Adverse events* Wound maceration, periwound blistering and exacerbation of pre-existing viral illnesses unrelated to burns. No instances of wound infection in the IG. In the CG there was a single case of exacerbation of a pre-existing viral disease, but no other EA		
			Both the IG and the CG returned to the burn center every 3–5 days to remove and reapply the NPWT and/or standard dressings until the wound was closed.	*Statistically significant scar thickness in the IG at 3 months (p = 0.018) but not at 6 months (p = 0.928).*		
				*IG 10 was suspended NPWT.*		
Frear et al., 2021 [26], Australia	N:101	ECA	The IG was treated with standard dressings consisting of Acticoat™ and Mepitel™ in combination with NPWT.	*Average re-epithelialization time:*	Adjunctive NPWT with conventional dressings is a cost-effective treatment for small-area burns in children.	Low
			NPWT → −80 mmHg.	IG: 8 days (IQR 7–11)		
			NPWT → −40 mmHg (extremities children under 12 months).	CG: 10 days (IQR 8–14)		
				*The mean reduction per participant in the intervention group (IG) was 3.19 (95% CI: 0.43–5.95 days).*		
				*Grafts:*		
				IG: 1 (2.1%)		
				CG: 4 (7%)		
	G: 47		The dressing changes in both groups were performed every 3–5 days until they were discharged or referred to scar treatment.	*Derivation treatment scars:*		
				IG: 10.6%		
				CG: 26.3%		
				*Surgery:*		
	CG: 54		The CG received only standard dressings.	IG: 2		
				CG: 7		
				*Total costs:*		
				NPWT: USD 90,369 (IQR 670.68–1234.74)		
				Conventional dressings USD 1669.01 (IQR 659.06–3269.16).		
Poulakidas et al., 2016 [32], USA	N:3	Retrospective single-center case series study	After debridement, the NPWT KCI VAC^®^ was applied together with the Acticoat^TM^ dressing (Smith and Nephew). To complete the healing process, patients were sent home with an application of silver sulfadiazine for 2–3 weeks.	Average length of stay in hospital: 9 days.	The three children who suffered frostbite did not require amputations thanks to NPWT.	Low
				No amputation with NPWT.		
				The duration of NPWT was 5 days in one case and 6 days in two cases.		
Yuan et al., 2016 [33], China	N: 73	Retrospective single-center case-control study	Controls: Surgical debridement and change of bandages and skin graft.	*100% successful NPWT and faster granulation.*	Reduction of dressing changes with vacuum sealing drainage (VSD) and efficient drainage improves tissue growth and leads to less scar formation.	Low
				*Bacteria:*		
				IG: 51, but no impact on 50 of 53.		
	Cases: 53			CG: 17.		
			Cases: Wound surface debridement before NPWT. Sponge placement on the wound, sealed with a semipermeable membrane. After 7–10 days an artificial dermis (PELNAC) was implanted in the granulation tissue.	*Grafts:*		
				Casos: 25		
	Controls: 20			Controls: 11		
				*Skin grafted into the artificial dermis was smoother and brighter, with a thicker and more elastic texture, and a lighter scar with NPWT (p < 0.05)*		
Zheng et al., 2019 [34], China	N: 64 Cases: 32 Controls: 32	Prospective before-after randomized controlled trial	Simple debridement was performed in the patients of two groups. IG: Micro-negative pressure with negative pressure material replaced every 3 to 5 days. CG: Conventional dressing change every other day.	*Graft survival rate:* Significantly higher in the IG. *Healing time:* Shorter in the IG. *Scar quality:* Better cosmetic and functional outcomes (fewer hypertrophic scars, improved elasticity) in the IG. *Complications:* Lower incidence of infection and necrosis in the IG.	Compared to conventional dressing changes, the use of micro-negative pressure therapy in children with small-area deep partial-thickness burns can significantly enhance the wound healing rate and surgical skin graft success, reduce the incidence of wound infections, shorten the overall healing time, and improve the quality of wound recovery.	Low

**Abbreviations:** N: number of study participants; IG: intervention group; CG: control group; RCT: randomized clinical trial; NPWT: negative pressure wound therapy; IQR: interquartile range; CI: confidence interval; AE: adverse effects; TBSA: total body surface area.

**Table 5:** Characteristics of participants.

Authors	Age	Sex *n* (%)	Type of Wound	Total Body Surface (TBSA) (%)	Part of the Body Affected
Ren et al., 2017 [31], USA	9.34 ± 2.03 years	Boys: 58.6%	*n* = 22 burns	27.62–9.83% (range 1–95%)	N/A
			*n* = 2 scalds		
			*n* = 4 contact		
			*n* = 7 electrical burns		
		Girls: 41.4%	*n* = 3 flame		
			*n* = 6 burning clothes		
			*n* = 7 Stevens–Johnson syndrome		
Frear et al., 2020 [25], Australia	<17 years	Boys: 58.4%	Burns:	<5% TBSA	Arms, legs, chest, torso, back, genitals and buttocks.
			*Scald:*		
			*n* = 28 IG		
			*n* = 35 CG		
			*Contact:*	IG: 1.5% (IQR 1–2)	
		Girls: 41.6%	*n* = 17 IG		
			*n* = 18 CG		
			*Flame:*	CG: 1% (IQR 1–2)	
			*n* = 2 IG		
			*n* = 1 CG		
Frear et al., 2021 [26], Australia	<17 years	Boys: 58.4%	Burns:	<5% TBSA	Arms, legs, chest, torso, back, genitals and buttocks.
			*Scald:*		
			*n* = 28 IG		
			*n* = 35 CG		
			*Contact:*	IG: 1.5% (IQR 1–2)	
		Girls: 41.6%	*n* = 17 IG		
			*n* = 18 CG		
			*Flame:*	CG: 1% (IQR 1–2)	
			*n* = 2 IG		
			*n* = 1 CG		
Hoeller et al., 2014 [30], Austria	8 ± 6 medium age	Boys: 60%	Burns from:	4.5% (3.0–12.0%), partial-thickness burns and full-thickness burn.	Head/face and neck; upper limbs and trunk; genital/gluteal region; and thighs, calves and feet.
			*n* = 20 flame		
	Less than 3 months.		*n* = 25 scalds	Deep partial-thickness burn, and full-thickness burn 4% (2.0–6.0%).	
		Girls: 40%	*n* = 2 chemical burn		
	Older than 24 years.		*n* = 1 hot air stream	TBSA: 3.5%.	
			*n* = 12 scar revision		
Poulakidas et al., 2016 [32], USA	16, 22, y 31 months	Boys: 66.7%	Freeze burns from 30 to 60 min exposure to temperatures between −12.6 and −18.1 °C.	N/A	Hands (unilateral or bilateral)
		Girls: 33.3%			
Yuan et al., 2016 [33], China	Cases: 5.5 ± 3.2	*Cases:*	*Abrasions from traffic accidents:*	N/A	N/A
		Boys: 66%	*n* = 46 cases		
		Girls: 34%	*n* = 18 controls		
			*Scalds:*		
	Controls: 6.2 ± 2.8		*n* = 5 cases		
		*Controls:*	*n* = 2 controls		
		Boys: 55%	*Infected wounds:*		
		Girls: 45%	*n* = 2 cases		
			*n* = 0 controls		
Zheng et al., 2019 [34], China	IG: 5.5 ± 2.8 CG: 5.8 ± 1.6	*Cases:* Boys: 56.25% Girls: 43.75% *Controls:* Boys: 62.5% Girls37.5%	Deep second-degree burns requiring surgical grafting	IG: (5.5 ± 2.2) % TBSA CG: (5.8 ± 1.6) % TBSA	Arms, legs, trunk and face.

**Table 6:** Summary of use of negative pressure therapy.

Authors	Mode	Pressure Measurements (mmHg)	Interface Layer or Foam	Treatment During the Intervention and/or Graft
Ren et al., 2017 [31], USA	Continuous	50 mmHg hasta −125 mmHg	N/A	First NPWT KCI VAC ^®^ and then partial-thickness skin graft.
Frear et al., 2020 [25], Australia	Continuous	80 mmHg and −40 mmHg for children under 12 months	IG: fiber mesh impregnated with nanocrystalline silver called Acticoat^TM^ (Smith and Nephew), and Mepitel^TM^ (Mölnlycke Health care), a silicone interface in combination with NPWT	NPWT Renasys Touch^TM^ (Smith and Nephew)
			CG: Acticoat^TM^ (Smith and Nephew), and Mepitel^TM^ (Mölnlycke Health care)	
Frear et al., 2021 [26], Australia	Continuous	80 mmHg and −40 mmHg for children under 12 months	IG: fiber mesh impregnated with nanocrystalline silver called Acticoat ^TM^ (Smith and Nephew), and Mepitel ^TM^ (Mölnlycke Health care), a silicone interface in combination with NPWT	NPWT Renasys Touch^TM^ (Smith and Nephew)
			CG: Acticoat^TM^ (Smith and Nephew), and Mepitel^TM^ (Mölnlycke Health care)	
Hoeller et al., 2014 [30], Austria	Continuous	−75 mmHg to −125 mmHg	Silicone layer and polyurethane film	Skin graft covered by NPWT
Poulakidas et al., 2016 [32], USA	N/A	N/A	NPWT KCI VAC ^®^ together with Acticoat ^TM^ dressing (Smith and Nephew)	NPWT KCI VAC^®^
Yuan et al., 2016 [33], China	N/A	−50 mmHg to −150 mmHg	N/A	First NPWT and then partial-thickness skin graft (PELNAC)
Zheng, et al., 2019 [34], China	Continuous	N/A	Silver sulfadiazine cream dressing in CG	N/A

## Data Availability

Data sharing is not applicable. No new data were created or analyzed in this study.

## Supplementary Materials

The following supporting information can be downloaded at: https://www.mdpi.com/article/10.3390/healthcare14020242/s1, File S1: PRISMA Checklist.

## References

[1] Ramírez J.E., Boswijk K., Morales J.G. (2019). Public Health and Burns Management in Emergencies within the Framework of the Knowledge and Information Society. Rev. Caribeña Cienc. Soc..

[2] Solís F.F., Domic C.C., Saavedra O.R., González M.A. (2014). Incidence and prevalence of burn injuries in children under the age of 20 years. Rev. Chil. Pediatr..

[3] Pereima M.J.L., Feijó R., da Gama F.O., Boccardi R.d.O. (2019). Treatment of burned children using dermal regeneration template with or without negative pressure. Burns.

[4] Asociación Española de Pediatría (2011). Quemaduras.

[5] Garnacho Saucedo G.M., Salido Vallejo R., Moreno Giménez J.C. (2020). Effects of solar radiation and an update on photoprotection. An. Pediatr. (Engl. Ed.).

[6] Hop M.J., Polinder S., van der Vlies C.H., Middelkoop E., van Baar M.E. (2014). Costs of burn care: A systematic review. Wound Repair Regen..

[7] Peck M.D. (2011). Epidemiology of burns throughout the world. Part I: Distribution and risk factors. Burns.

[8] Lumsden E.J., Kimble R.M., McMillan C., Storey K., Ware R.S., Griffin B. (2023). Protocol for a feasibility, acceptability and safety study of the PICO device (negative pressure wound therapy) in acute paediatric burns. BMJ Open.

[9] Pedrazzi N.E., Naiken S., La Scala G. (2021). Negative Pressure Wound Therapy in Pediatric Burn Patients: A Systematic Review. Adv. Wound Care.

[10] Holbert M.D., Kimble R.M., Jones L.V., Ahmed S.H., Griffin B.R. (2021). Risk factors associated with higher pain levels among pediatric burn patients: A retrospective cohort study. Reg. Anesth. Pain Med..

[11] Lumsden E., Kimble R., McMillan C., Storey K., Ware R.S., Griffin B. (2023). The feasibility of negative pressure wound therapy versus standard dressings in paediatric hand and foot burns protocol: A pilot, single-centre, randomised control trial. Pilot Feasibility Stud..

[12] Chan L.K.W., Lee K.W.A., Hung L.C., Lam P.K.W., Wan J., Vitale M., Huang P.P., Yi K. (2024). Treating hypertrophic scar, post-inflammatory hyperpigmentation, and post-inflammatory hypopigmentation with intense pulsed light. Skin Res. Technol..

[13] Rodríguez-Fuentes G., Romero Rodríguez T. (2022). Physiotherapy in scars. Review of current state. Cir. Plast. Ibero-Latinoam..

[14] McPhail S.M., Wiseman J., Simons M., Kimble R., Tyack Z. (2022). Cost-effectiveness of scar management post-burn: A trial-based economic evaluation of three intervention models. Sci. Rep..

[15] Canelos Moreno J.A., Williams Vargas L.N., Hidalgo Bermudez C.A. (2021). Burns in pediatrics. Therapeutic Perspectives. Polo Conoc..

[16] Hernández-Patiño I., Rossani G., De La Cruz V.J.A., Casado F.L., Trelles M.A. (2020). Treatmen of burns by heterologous rich platelet plasma (hRPP). About a pediatric case report. Cir. Plast. Ibero-Latinoam..

[17] Çelik E., Akelma H. (2023). Hydrogel burn dressing effectiveness in burn pain. Burns.

[18] Bairagi A., Griffin B., Banani T., McPhail S.M., Kimble R., Tyack Z. (2021). A systematic review and meta-analysis of randomized trials evaluating the efficacy of autologous skin cell suspensions for re-epithelialization of acute partial thickness burn injuries and split-thickness skin graft donor sites. Burns.

[19] Cheng Y., Zhou Z., Hu H., Li W., Liu Y., Xia Z., Deng C., Mao G., Yi L., Liu X. (2020). An Application of a Negative-Pressure Wound Dressing for Partial- or Full-Thickness Burn Wounds. Int. J. Low. Extremity Wounds.

[20] Kim J.-H., Lee D.-H. (2019). Negative pressure wound therapy vs. conventional management in open tibia fractures: Systematic review and meta-analysis. Injury.

[21] Song Y., Wang L., Yuan B., Shen H., Du L., Cai J., Chen H. (2021). Negative-pressure wound therapy for III/IV pressure injuries: A meta-analysis. Wound Repair Regen..

[22] Maitret-Velázquez R.M., Bizueto-Rosas H., Gómez-Calvo C.D., Pérez-González H.A., Moreno-Ramírez C.I., Hernández-Vázquez J.I. (2018). Uso de la terapia de presión negativa para manejo de heridas complejas. Rev. Mex. Angiol..

[23] Moher D., Liberati A., Tetzlaff J., Altman D.G. (2009). Preferred Reporting Items for Systematic Reviews and Meta-Analyses: The PRISMA Statement. PLoS Med..

[24] Higgins J.P., Savovic J., Page M.J., Sterne J.A. (2019). Revised Cochrane risk-of-bias tool for randomized trials (RoB 2). Cochrane Handbook for Systematic Reviews of Interventions.

[25] Frear C.C., Cuttle L., McPhail S.M., Chatfield M.D., Kimble R.M., Griffin B.R. (2020). Randomized clinical trial of negative pressure wound therapy as an adjunctive treatment for small-area thermal burns in children. Br. J. Surg..

[26] Frear C.C., Griffin B.R., Cuttle L., Kimble R.M., McPhail S.M. (2021). Cost-effectiveness of adjunctive negative pressure wound therapy in paediatric burn care: Evidence from the SONATA in C randomised controlled trial. Sci. Rep..

[27] Centro Cochrane Iberoamericano Traductores (2012). Manual Cochrane de Revisiones Sistemáticas de Intervenciones, Versión 5.1.

[28] Aromataris E., Lockwood C., Porritt K., Pilla B., Jordan Z. (2024). JBI Manual for Evidence Synthesis.

[29] Borenstein M., Hedges L.V., Higgins J.P.T., Rothstein H.R. (2010). A basic introduction to fixed-effect and random-effects models for meta-analysis. Res. Synth. Methods.

[30] Hoeller M., Schintler M.V., Pfurtscheller K., Kamolz L.-P., Tripolt N., Trop M. (2014). A retrospective analysis of securing autologous split-thickness skin grafts with negative pressure wound therapy in paediatric burn patients. Burns.

[31] Ren Y., Chang P., Sheridan R.L. (2017). Negative wound pressure therapy is safe and useful in pediatric burn patients. Int. J. Burn. Trauma.

[32] Poulakidas S.J., Kowal-Vern A., Atty C. (2016). Pediatric Frostbite Treated by Negative Pressure Wound Therapy. J. Burn. Care Res..

[33] Yuan X.G., Zhang X., Fu Y.X., Tian X.F., Liu Y., Xiao J., Qiu L. (2016). Sequential therapy with “vacuum sealing drainage-artificial dermis implantation-thin partial thickness skin grafting” for deep and infected wound surfaces in children. Orthop. Trau-Matology Surg. Res..

[34] Zheng X.P., Chen J., Chen T.S., Jiang Y.N., Shen T., Xiao S.C., Hu X.Y. (2019). Preliminary effect observation on the application of micro-negative pressure in children with small-area deep partial-thickness burn. Chin. J. Burn..

[35] Munn Z., Barker T.H., Moola S., Tufanaru C., Stern C., McArthur A., Stephenson M., Aromataris E. (2020). Methodological quality of case series studies. JBI Evid. Synth..

[36] Moola S., Munn Z., Tufanaru C., Aromataris E., Sears K., Sfetic R., Currie M., Lisy K., Qureshi R., Mattis P. (2020). Systematic reviews of etiology and risk. JBI Man. Evid. Synth..

[37] Taha A.G., Sayed A.I., Ahmed O.M. (2023). Role of negative pressure wound therapy in the management of surgically treated diabetic foot infections: A randomized controlled trial. Egypt. J. Surg..

[38] Srivastava V., Meena R.N., Pratap A., Verma A.K., Ansari M.A., Mishra S.P. (2022). Effect of negative pressure wound therapy on wound thermometry in diabetic foot ulcers. J. Fam. Med. Prim. Care.

[39] Maranna H., Lal P., Mishra A., Bains L., Sawant G., Bhatia R., Kumar P., Beg M.Y. (2021). Negative pressure wound therapy in grade 1 and 2 diabetic foot ulcers: A randomized controlled study. Diabetes Metab. Syndr. Clin. Res. Rev..

[40] Anjum W., Ali S.Z., Mumtaz M., Imran M., Siddique H., Zia H. (2022). Comparison of Vacuum Assisted Closure (VAC) Therapy Versus Conventional Dressing in the Management of Diabetic Foot Ulcer. Pak. J. Med. Health Sci..

[41] Adham A.H.M., Talaat A.A., Hana I.N., Abdelmoaty K.F. (2022). The value of negative pressure wound therapy in comparison with the conventional dressing on the postoperative wound healing in diabetic foot patients. Egypt. J. Surg..

[42] Kasukurthi R., Borschel G.H. (2010). Simplified Negative Pressure Wound Therapy in Pediatric Hand Wounds. HAND.

[43] Argenta L.C., Morykwas M.J. (1997). Vacuum-assisted closure: A new method for wound control and treatment: Clinical experience. Ann. Plast. Surg..

[44] Roohaninasab M., Nobari N.N., Ghassemi M., Behrangi E., Jafarzadeh A., Sadeghzadeh-Bazargan A., Goodarzi A. (2024). A systematic review of procedural treatments for burn scars in children: Evaluating efficacy, safety, standard protocols, average sessions and tolerability based on clinical studies. Int. Wound J..

[45] Li Z., Yu A. (2014). Complications of negative pressure wound therapy: A mini review. Wound Repair Regen..

[46] Sahin I., Eski M., Acikel C., Kapaj R., Alhan D., Isik S. (2012). The role of negative pressure wound therapy in the treatment of fourth-degree burns. Trends and new horizons. Ann. Burn. Fire Disasters.

[47] Zhao J., Shi K., Zhang N., Hong L., Yu J. (2024). Assessment between antiseptic and normal saline for negative pressure wound therapy with instillation and dwell time in diabetic foot infections. Sci. Rep..

[48] Mushin O.P., Bogue J.T., Esquenazi M.D., Toscano N., Bell D.E. (2017). Use of a home vacuum-assisted closure device in the burn population is both cost-effective and efficacious. Burns.

[49] Rai S., Mahendru V., Richaria A., Musa O., Rizvi A.H. (2019). Comparative study of efficacy of negative pressure wound therapy versus conventional dressing in open wounds. Natl. J. Med. Allied Sci..

[50] Johari H.G., Kazemzadeh G.H., Modaghegh M.-H.S., Ravari H., Sangaki A., Shahrodi M.V., Vatanchi A.M. (2013). Comparision of vacuum-asisted closure and moist wound dressing in the treatment of diabetic foot ulcers. J. Cutan. Aesthet. Surg..

[51] Apelqvist J., Armstrong D.G., Lavery L.A., Boulton A.J.M. (2008). Resource utilization and economic costs of care based on a randomized trial of vacuum-assisted closure therapy in the treatment of diabetic foot wounds. Am. J. Surg..

[52] Ferrari A.J., Santomauro D.F., Aali A., Abate Y.H., Abbafati C., Abbastabar H., ElHafeez S.A., Abdelmasseh M., Abd-Elsalam S., Abdollahi A. (2024). Global incidence, prevalence, years lived with disability (YLDs), disability-adjusted life-years (DALYs), and healthy life expectancy (HALE) for 371 diseases and injuries in 204 countries and territories and 811 subnational locations, 1990–2021: A systematic analysis for the Global Burden of Disease Study. Lancet.

